# Mechanical Properties of Hybrid Graphene Nanoplatelet-Nanosilica Filled Unidirectional Basalt Fibre Composites

**DOI:** 10.3390/nano11061468

**Published:** 2021-06-01

**Authors:** Ummu Raihanah Hashim, Aidah Jumahat, Mohammad Jawaid

**Affiliations:** 1 Faculty of Mechanical Engineering, Universiti Teknologi MARA (UiTM), Shah Alam 40450, Selangor, Malaysia; ummuraihanahhashim@gmail.com; 2 Institute for Infrastructure Engineering Sustainable and Management (IIESM), Universiti Teknologi MARA, Shah Alam 40450, Selangor, Malaysia; 3 Department of Biocomposite Technology, Institute of Tropical Forestry and Forest Products, Universiti Putra Malaysia (UPM), Serdang 43400, Selangor, Malaysia

**Keywords:** basalt fibre, graphene nanoplatelets (GNP), nanosilica, hybrid filler, epoxy polymer, mechanical properties

## Abstract

Basalt fibre (BF) is one of the most promising reinforcing natural materials for polymer composites that could replace the usage of glass fibre due to its comparable properties. The aim of adding nanofiller in polymer composites is to enhance the mechanical properties of the composites. In theory, the incorporation of high strength and stiffness nanofiller, namely graphene nanoplatelet (GNP), could create superior composite properties. However, the main challenges of incorporating this nanofiller are its poor dispersion state and aggregation in epoxy due to its high surface area and strong Van der Waals forces in between graphene sheets. In this study, we used one of the effective methods of functionalization to improve graphene’s dispersion and also introducing nanosilica filler to enhance platelets shear mechanism. The high dispersive silica nanospheres were introduced in the tactoids morphology of stacked graphene nanosheets in order to produce high shear forces during milling and exfoliate the GNP. The hybrid nanofiller modified epoxy polymers were impregnated into BF to evaluate the mechanical properties of the basalt fibre reinforced polymeric (BFRP) system under tensile, compression, flexural, and drop-weight impact tests. In response to the synergistic effect of zero-dimensional nanosilica and two-dimensional graphene nanoplatelets enhanced the mechanical properties of BFRP, especially in Basalt fibre + 0.2 wt% GNP/15 wt% NS (BF-H0.2) with the highest increment in modulus and strength to compare with unmodified BF. These findings also revealed that the incorporation of hybrid nanofiller contributed to the improvement in the mechanical properties of the composite. BF has huge potential as an alternative to the synthetic glass fibre for the fabrication of mechanical components and structures.

## 1. Introduction

The use of Fibre Reinforced Polymer (FRP) composites has been expanded from time to time for numerous strengthening applications in various industries. The fibre reinforcement in polymer composites include materials either from synthetic fibres (carbon, glass, aramid), renewable sources or natural fibres (jute, basalt, kenaf) and by-products from food crops and recycle wastes (paper, wood), which could provide stiffness and strength of the final materials to be used in a structure for different applications. Fibres are generally categorised into two main types; natural and synthetic fibres where are then further subcategorised based on their origin. Currently, the demand for the use of natural fibres has been explored as an alternative replacement for synthetic fibres to give benefits to the environment and to enhance sustainability. Although natural fibres have a relatively lower strength compared to synthetic fibres, certain modifications by chemical treatments, as well as the development of the system, if proven could prevail in their limitations.

Fibre Reinforced Polymers (FRPs) are composite materials that consist of short or continuous fibres that are bonded together by a polymer matrix. Fibres, which are the load-bearing constituent, provide high stiffness and strength in the polymer composite. It is a natural or synthetic substance that acts as a reinforcement to enhance the strength and elasticity of plastics in the form of continuous filaments or discrete elongated pieces as similar to pieces of thread. FRP composites’ mechanical efficiency is dependent upon the interface provided by the fibre-matrix along with stress transfer, in which stress is transferred to the fibre from the matrix. Materials derived from natural products are emerging as potential substitutes for petroleum-based plastics for a variety of applications. The properties of Natural Fibre Reinforced Polymer Composite (NFRPC) vary with fibre types and fibre sources as well as fibre structures. Natural fibres received considerable attention in various applications owing to their properties, such as relatively low weight, low cost, less damage to processing equipment and good relative mechanical properties such as tensile modulus and flexural modulus and biodegradability [1]. Nowadays, natural fibres are comprehensively explored as they have the potential to be used as a component of composite materials for broad applications within the textile, building, plastic and automotive industries. In the building industry, the performance of natural fibres that allow insulation properties higher than current materials makes the interest in natural fibres mostly economical and technical. Meanwhile, within the automotive industry, the use of natural fibres at the industrial level improves the environmental sustainability of the parts being constructed [2,3,4].

Basalt stone has been used since antiquity in construction and nowadays, as the technology has developed, basalt stone is melted and processed to make basalt fibre. Basalt fibre (BF) is a mineral-based natural fibre that originated from the solidification of hot magma flows rising from the volcanoes or cracks in the Earth’s crust [1,5,6,7]. It is primarily used in high-end infrastructural and civil applications such as for insulation walls, chimneys, beams, and others. Recent progress on the development of basalt fibre has occurred by hybridising this fibre with other material to extend its potential for construction and automotive purposes. Ribeiro et al. [8] combined basalt and carbon fibres and evaluated the tensile properties of this hybrid material, and it was found that this combination worked efficiently based on the high strength value obtained. Khandelwal and Ree [9] summarized the recent development of basalt fibre reinforced composites by reviewing the methods to enhance the fibre-matrix interface of this fibre in cement composites and polymer composites. They suggested that the best way, to date, is by using the nanofillers to strengthen the BF in polymer composites or to improve the fibre–matrix interface. Toth [10] has investigated the effect of nanofiller incorporation into BF epoxy composite in the mechanical and tribology properties. The findings showed that the addition of the fillers (polytetrafluoroethylene (PTFE), polyoxymethylene (POM) or molybdenum disulphide (MoS2)) contribute to the improvement in hardness, strength and coefficient of friction of the BFRP composite. In another work, Marquis et al. [11] and Wang et al. [12] discovered that the surface treatment and mixing process efficiency are the key points dictating the performance of nanofillers.

In terms of compatibility with epoxy resin, nanosilica (NS) has been found to be the best nanofiller to be used. Wei et al. [13] found that the addition of NS to the resin improved the tensile strength of the system. Jumahat et al. [14] found that the presence of NS in the epoxy resin improved the ductility and enhanced the energy absorption without reducing the deformation or failure of the system better than carbon nanotubes (CNT) and nanoclays. This is due to the homogeneous dispersion of silica nanoparticles even at higher loading. The impact and fracture toughness properties of BF epoxy composite with NS has been studied by Demirci [15], and it was concluded that the NS existence improves the fracture mechanism as well as the interfacial bonding between fibre and matrix by retarding the crack propagation during impact.

The use of carbon-based nanofillers such as CNT and graphene has captured researchers’ attention owing to their excellent properties such as high aspect ratio, high mechanical stiffness, high strength, and thermal conductivity [11,16,17]. The use of graphene in epoxy has brought a new class of nanocomposites for advanced engineering applications. However, graphene is not suitable to be dispersed in epoxy just by simple mixing due to the strong van der Waals force and pi-stacking between graphene sheets, which make them tend to aggregate in the epoxy matrix [12,18,19]. It is crucial to ensure the homogeneous dispersion to be achieved to get the maximum performance of the finish materials. The covalent functionalisation of graphene with chemicals could improve the dispersion and reduce the aggregation. However, this method might degrade or change the selected properties of graphene. The use of solvents has been investigated and accepted as the simplest method to disperse graphene [16,20,21,22,23,24,25,26,27]. Other than that, many studies have been conducted to develop an alternative method to improve the dispersion and mechanical properties of graphene in the polymer [28,29,30,31,32]. According to Garcia et al. [29], proper CNT dispersion and improved interfacial properties could be achieved by the introduction of nano/microstructures to the CNT. He et al. [28] introduced alumina to CNT to form a nano-micro hybrid structure and studied the effect of CNT length diameter on the organization modes of the hybrid structure.

For this study, NS with a known good dispersion state was used to help the dispersion process of GNP using the solvent exchange and milling method to improve the graphene’s dispersion to enhance the final properties of the composite by executing the mechanical shear force to the stacked graphene sheets during the milling process. Thus, the graphene stacks were broken into smaller graphene layers, which dispersed better in the matrix. The motivation behind the development of hybrid nanofiller materials is to achieve an improvement of the nanocomposites through the combination and synergistic effect of both materials and employed in the natural basalt fibre reinforced polymer composites. The improvement mechanism of hybrid filler in terms of mechanical properties may be due to shape formation and dimensional structure, the interaction between nanofillers, optimum weight ratio, proper processing technique and good filler dispersion.

## 2. Materials and Methods

This study used Miracast 1517 A/B as the matrix in the composite owing to its versatility in various high-performance applications. It was manufactured and supplied by Miracon (M) Sdn. Bhd, Selangor, Malaysia. It is a low viscosity Diglycidyl Ether of Bisphenol-A (DGEBA) epoxy, which was designed for the production of composite laminates that meet all the high-performance properties of a composite structure. The amine-curing hardener was added to the epoxy with a ratio of 100:30 (epoxy: hardener). The unidirectional BF was used in the fabrication of BFRP nanocomposites. This study used the fabric form of unidirectional BF supplied by Innovative Pultrusion Sdn. Bhd., Negeri Sembilan, Malaysia, with a diameter of 7 µm and a density of 2.00 g/cm^3^. It has a higher tensile strength and elastic modulus than E-glass fibre; 4.8 GPa and 78 GPa, respectively. Glass fibre (GF) fabric was used as a comparison and benchmarking material to the BF. GF composite was fabricated to compare the performance of natural fibre, basalt to the synthetic fibres and glass fibre. The unidirectional GF fabric was supplied by Innovative Pultrusion Sdn. Bhd., Negeri Sembilan, Malaysia in the form of a 500 mm width × 27 kg roving roll. GF used in this study is a unidirectional continuous E-glass fibre with a diameter of 10 µm and a density of 2.04 g/cm^3^. It has a tensile strength and elastic modulus of 3.45 GPa and 72 GPa, respectively. The graphene nanoplatelets (GNP) used in this study were XGnP-Graphene Nanoplatelets Grade M5 in the form of black granules manufactured by XG Sciences, Lansing, MI, USA with an average thickness of 6–8 nm, average particle diameter of 5 µm, and a typical surface area of 120 to 150 m^2^/g. Like other nanoscale materials, GNP requires special handling and processing. GNP comes in a granular form, and it must be fully dispersed to exhibit their optimal properties. In this study, the solvent-exchange method was used to enhance the GNP dispersion. Another nanofiller used was Nanosilica (NS). NS used in this study was Nanopox F400 gel manufactured by Evonik Industries AG, Essen, Germany. It is a high performance, versatile, silica-reinforced bisphenol-A based epoxy resin for use in fibre composites. It consists of modified SiO_2_ with a very small particle size of 20 nm (which is good to penetrate the tightly meshed fibres) and narrow particle size distribution (maximum diameter of 50 nm). Despite the high silica content of 40 wt%, this nanosilica gel has a comparatively low viscosity due to agglomerate free colloidal dispersion of the nanoparticles in the resin. This type of NS gel is suitable to be used with any types of commercial epoxy resins and hardeners, as well as for injection and infusion process due to its low viscosities.

### 2.1. Fabrication of Fibre Reinforced Polymer Hybrid Nanocomposites

**Figure 1:**
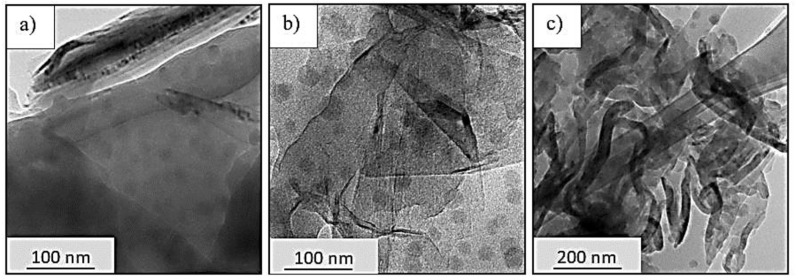
TEM Images of 0.2 wt% GNP in (**a**) 5 wt% NS, (**b**) 15 wt% NS, and (**c**) 25 wt% NS.

The selection of nanofiller loading is based on a literature survey and a preliminary experiment. As for the GNP modified epoxy system, the maximum 6 wt% of GNP was conducted beforehand; however, the optimum loading is found to be 0.3 wt%, where at this value the maximum enhancement could be achieved in the finish composite material. In order to select the optimum nanosilica content for the hybrid nanofiller system, the preliminary test was conducted beforehand by incorporating an average of 0.2 wt% GNP in 5, 15 and 25 wt% NS to identify the best nanosilica portion in the hybrid system. Figure 1 shows the TEM images on the dispersion state of the hybrid nanofiller in the epoxy resin. It can be observed that there is no significant agglomeration in the hybrid system with 0.2 wt% GNP in 5 wt% and 15 wt% NS as shown in Figure 1a,b. However, the incorporation of 0.2 wt% GNP in 25 wt% NS shows serious agglomeration at almost all areas, as in Figure 1c. This condition occurred due to improper blending during the fabrication because of the high viscosity of the system. Hence, based on these results, 15 wt% NS was selected to be hybridized with GNP at 0.1, 0.2 and 0.3 wt% with a longer milling time in order to identify the hybridization effect of different GNP loading.

**Figure 2:**
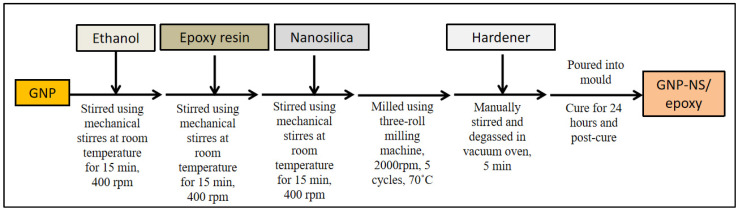
The schematic diagram for the fabrication of hybrid GNP-NS/epoxy composites.

The fabrication of GNP/epoxy and hybrid systems used ethanol as the solvent to disperse the GNP in the epoxy resin. Ethanol was selected as the solvent due to its low boiling point. However, owing to its high surface tension, which is 22.1 mN/m at 30 °C, the direct exfoliation of GNP in ethanol is not suitable. High surface tension has a negative effect on the dispersion of the GNP. Hence, the GNP first underwent a solvent exchange process with N-methyl-2-pyrrolidone (NMP) to form a relatively stable dispersion in ethanol. For the hybrid system, higher nanosilica content leads to higher viscosity of the polymer nanocomposites, leading to difficulties in the mixing process. The control specimens of BFRP and GFRP with NS or GNP were prepared at the highest filler content (25 wt% for NS, and 0.3 wt% for GNP). These selections were made based on the preliminary studies where the incorporation of higher than these percentages generates difficulties during the mixing process due to their high viscosity. Figure 2 shows the processing steps used to produce the hybrid nanofiller epoxy composites. Initially, the solvent exchange process was implemented to stabilize the GNP dispersion before mixing with NS. Then, the GNP was dispersed in ethanol and stirred for 15 min at 400 rpm before adding the epoxy resin. After that, the NS was added into the mixture and milled for five cycles to ensure the good dispersion of nanofillers in the epoxy resin. Once completed, the hardener was added, and the mixture was stirred and degassed in a vacuum oven before being poured into the mould and left to cure for 24 h. The designation of fabricated FRP composite systems was tabulated in Table 1.

The FRP composite laminates were fabricated using the hand-layup technique. Firstly, the unidirectional fibre fabric was cut according to the required size. Then, an aluminium plate with the polytetrafluoroethylene (PTFE) release films placed on it was used as the base plate. PTFE particles provided excellent dry lubrication for the release of a cured composite. Next, the unmodified or nanomodified epoxy resin with the hardener prepared previously was applied onto the PTFE release film and spread evenly in all directions. After that, a layer of unidirectional fibre fabric was placed onto the epoxy resin. Both steps were repeated until the required thickness was achieved by placing the fibre and spreading the epoxy resin alternately layer by layer. The thickness of the specimen depends on the conducted test. Once the required thickness was achieved, a perforated release film was put on top of the laminate followed by an absorption fabric. The perforated release film allowed excessive resin from the laminate to pass through, before being absorbed by the absorption fabric. Finally, a vacuum bagging film was placed on the laminate and was pressed firmly against the sealant tape to provide a vacuum-tight system. A vacuum bagging film was carefully spread over the laminate to ensure no wrinkles would form when the vacuum was applied, as wrinkles would affect the surface finish of the laminate. The laminate was vacuumed for one hour to remove the air-trapped bubbles and excessive resin and was left to cure at room temperature for 24 h. After 24 h cured at room temperature, the FRP composite laminates were then post cured from 60 °C to 120 °C for 14 h through stepwise increases of 10–20 °C every 2 h. This process is essential to avoid the development of internal stresses within the casting as well as improper curing of the composites. 

### 2.2. Physical Test

#### 2.2.1. Density

In determining the density of the specimens, a density balance was used based on Archimedes principles in distilled water, according to ASTM D792. The average values were taken from five specimens for each system. The density of the FRP composites were measured to identify the differences before and after nanofiller incorporation. The addition of nanosilica and GNP to epoxy is expected to increase the composite density as these nanofillers have higher density than epoxy resin.

#### 2.2.2. Determination of Fibre Volume Fraction by Acid Digestion Method

In the acid digestion method, three specimens for each system were used to get the average measurement. Initially, each specimen was weighed to get its density using an analytical balance in accordance with ASTM D792. Each specimen was placed in a beaker containing 30 mL of 70% nitric acid. Then, the beakers were placed on the hot plate and heated up to 70 °C for an hour to completely digest the matrix. The matrix is considered to be fully digested when there are no traces of matrix/reinforcement laminate combination. After that, the content of each beaker was filtered under a vacuum, and the remaining reinforcement was washed three times with distilled water, followed by final washes with acetone to improve drying time. Finally, the specimens were dried in an oven at 100 °C for one and a half hours and cooled to room temperature in a desiccator before being weighed to the nearest 0.001 g.

### 2.3. Mechanical Tests

#### 2.3.1. Tensile Test

The tensile test was performed on the FRP nanocomposites specimen following ASTM D3039. The rectangular specimens with a dimension of 250 mm length × 15 mm width × 3 mm thickness were tested using the INSTRON 3382 Universal Testing Machine 100 kN load cell (Instron, Norwood, MA, USA). A clip-on extensometer of 25 mm gauge length was attached to the tested specimen to record the elongation data at the crosshead speed of 2 mm/min. These data were logged into computer software for analysis. Five specimens were tested for each FRP composites system.

#### 2.3.2. Compression Test

A static uniaxial compression test was conducted on the FRP nanocomposite specimens according to ASTM D3410. A rectangular specimen with a dimension of 110 mm length × 10 mm width × 3 mm thickness was prepared for this test. The compression test was conducted using an Universal Testing Machine INSTRON 3382 100 kN load cell (Instron, Norwood, MA, USA) with a special rig designed and fabricated according to the standard to suit the requirements of the testing machine. Five specimens for each FRP system were tested at a suggested crosshead speed of 1 mm/min.

#### 2.3.3. Flexural Test

The flexural test for basalt and glass FRP nanocomposites were conducted using ASTM D790 with specimen dimensions of 80 mm length × 15 mm width × 3 mm thickness and support spans of 48 mm. An INSTRON Universal Testing Machine 100 kN load cell (Instron, Norwood, MA, USA) with the three-point bending fixtures was used to apply force at midspan at the crosshead speed of mm/min. Five specimens were tested for each FRP composites system.

#### 2.3.4. Drop Weight Impact Test

The drop weight impact test was conducted according to ASTM D7136 using an INSTRON Dynatup 8250 Drop Weight Impact Tester, Instron, Norwood, MA, USA. Specimens with dimensions of 50 mm length × 50 mm width × 5 mm thickness were used in this test. A drop tower with a 16 mm hemispherical tip impactor was used with a weight of 5.5 kg, a drop height of 0.8 m, and a gravity acceleration of 9.81 m/s^2^, resulting in kinetic energy of 43.164 J. Five specimens were tested for each composite system.

### 2.4. Damage Evaluation

The damaged surface of the specimen after testing was examined using several surface metrology techniques such as optical microscopy and Scanning Electron Microscopy (SEM). These are important to characterise the failure modes of the composite materials. Information such as the origin of the crack propagation direction and the types of fracture could be identified by observing the fractured surface of the tested specimen. The fractured area on the specimen was sectioned and mounted onto the suitable holders and then coated with a thin layer of platinum using a Sputter Coater POLARON SC7620, Quorum Technologies Ltd, Kent, UK. The specimen was coated to provide the conducting surface and enhance the electron emission on the specimen surface. The specimen was then placed onto the aluminium stubs using carbon adhesive tape and was later put into the vacuum chamber of the SEM at a suitable accelerating voltage and resolution. 

## 3. Results and Discussion

The mechanical performance of the FRP composites were determined using tensile, compression, flexural and drop-weight impact tests. The effects of nanofiller hybridisation on basalt and glass FRP composites were investigated and the mechanical properties of the natural and the synthetic fibres were compared. SEM was used to study the fracture behaviour and failure mechanisms of the damaged specimens.

### 3.1. Physical Properties

#### 3.1.1. Density

The density of the FRP nanocomposites system was measured using the Archimedes principle. The result shows that BFRP composites have a lower density than GFRP with the average density of neat BFRP and GFRP composites being 1.552 g/cm^3^ and 1.613 g/cm^3^, respectively. The incorporation of both nanosilica and GNP filler increases the density of the FRP system. The density of the FRP nanocomposites is summarized in Table 2. Table 2 shows that as the nanofiller content increased, the density of the composite also increased. The incorporation of GNP nanofiller in the FRP composite system show lower density values as compared to the FRP composite with nanosilica as the content of the GNP used is very small. The inclusion of maximum 25 wt% nanosilica increases the density by 6% for BFRP, and 5% for GFRP composites and the inclusion of maximum 0.3 wt% GNP increase the density by 0.6% and 0.7%, respectively, as compared to an unmodified system. Meanwhile, the inclusion of 0.3 wt% GNP in hybrid filler increases the density by 1% for BFRP and 0.4% for GFRP composites.

#### 3.1.2. Fibre Volume Fraction

In order to determine the fibre volume fraction of the BFRP and GFRP composites in this study, the acid digestion method was used. The density measured using the analytical balance was used to calculate the fibre and matrices, as well as void volume fraction, as indicated in Table 3. The results show that both the BFRP and GFRP composites have a similar amount of fibre volume fraction. Good specimen quality was fabricated in this study as the void volume fraction on the composite was small. It can be deduced that the implementation of the vacuum bagging method was efficient at compacting the fibre reinforcement under vacuum pressure in order to produce specimens with a high fibre volume fraction, uniform thickness and low voids content. A good composite specimen should have less than 5 vol% voids, whereas more than 10 vol% voids shows poorly fabricated composite specimens. Nevertheless, the GFRP specimens displayed higher void content compared to BFRP composite.

### 3.2. Mechanical Properties

#### 3.2.1. Tensile Properties

**Figure 3:**
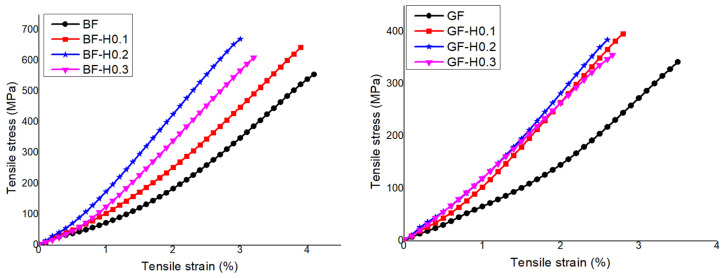
Typical tensile stress-strain response of GFRP and BFRP embedded with hybrid nanofiller.

Figure 3 displays the typical tensile stress-strain response of BFRP and GFRP embedded with hybrid nanofiller. According to the results tabulated in Table 4, the tensile modulus and strength of BF-H0.1 were increased by 56%, BF-H0.2 by 68%, and BF-H0.3 by 49%, respectively, to compare with unmodified BFRP and GFRP. The increments in tensile strength were 16%, 21%, and 10% for BF-H0.1, BF-H0.2, and BF-H0.3, respectively. From the results, it can be seen that the BF-H0.2 exhibited an optimum reinforcement effect, as the further increase of hybrid nanofiller led to a decrease in tensile modulus and strength. This figure also shows a significant enhancement in tensile modulus and strength of GFRP composite, which contributed by the addition of hybrid nanofiller. GF-H0.2 showed the highest tensile modulus with 81% increment, followed by GF-H0.1 with 53% and GF-H0.3 with 26%, respectively, compared to the unmodified GFRP. A similar trend was observed in terms of the tensile strength of the GFRP composite where GF-H0.1 was enhanced by 16%, GF-H0.2 by 12%, and GF-H0.3 by 4%, respectively. From these results, it can be deduced that the GF-H0.1 as the optimum hybrid system for GFRP, in which the modulus and strength increment was the highest. The effect of hybrid nanofiller on the GFRP was not very significant as the GNP content in the hybrid nanofiller increased. This could be because glass fibres have lower moisture resistance as compared to basalt fibres and the incorporation of a higher amount of GNP in hybrid nanofiller into the epoxy matrix may have hindered the wetting process and caused less matrix to be wetted on the fibres [33,34]. Hence, more resin is needed to properly adhere the matrix to the fibre, which results in a more significant improvement in tensile strength. Both FRP systems show a reduction in the tensile strain at the break, which is common as a result of rigid nanofiller incorporation into the epoxy. All of these findings show a positive and promising synergistic effect of hybrid nanofiller in terms of tensile properties. The finding showed that the FRP with hybrid nanofiller exhibited the best reinforcement effect in tensile properties compared to other single nanofillers. This is owing to the unique three-dimensional nanofiller structure and inherent properties of GNP and NS, which reduced the stress concentration and led to higher tensile properties [35].

**Figure 4:**
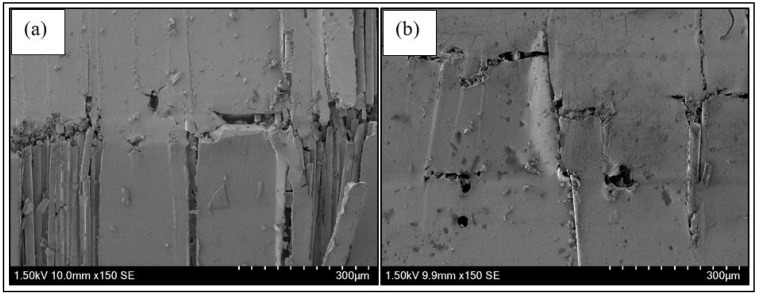
SEM micrographs showing the tensile damage specimens of (**a**) unmodified BF and (**b**) BF-H0.1 composites.

A comparative macroscopic and microscopic observation of the hybrid nanofiller modified composite specimens was also conducted to investigate the effects of nanofiller addition in the composite specimens. The presence of hybrid nanofiller in the FRP composites resulted in less crack propagation of the matrix as displayed in Figure 4. From the SEM micrographs, it was concluded that the failure was mainly caused by matrix cracking that has led to fibre breaking in unmodified specimens, while less matrix cracking was observed with the presence of nanofiller. This is due to the nanofiller existence that improved the matrix toughness and reduced the matrix dominated failure and expected to be experienced by other systems with GNP and NS nanofiller owing to the rigid filler characteristic that enhances the matrix toughness [36].

#### 3.2.2. Compressive Properties

**Figure 5:**
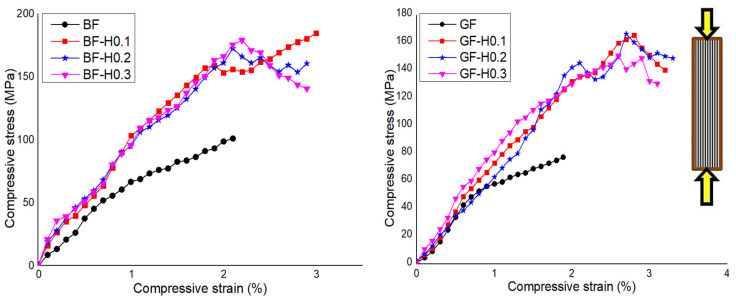
Typical compressive stress-strain response of GFRP and BFRP embedded with hybrid nanofiller.

The effect of nanofiller hybridisation on the compressive properties of BFRP and GFRP composites are investigated and discussed. Figure 5 shows the typical compressive stress-strain response of BFRP and GFRP embedded with a hybrid nanofiller. From the curves, it can be seen that the addition of hybrid nanofiller remarkably increased the compressive properties of both FRP composites. As summarised in Table 5, comparing the hybrid system to the unmodified BFRP, a significant improvement can be observed in compressive modulus of BF-H0.1, BF-H0.2 and BF-H0.3 by 64%, 69%, and 57%, respectively. The presence of hybrid nanofiller also increased the compressive strength by 72% for BF-H0.1, 61% for BF-H0.2, and 67% for BF-H0.3. For instance, the GF-H03 exhibited the highest compressive modulus with a 41% increment compared to that of unmodified GFRP. This is followed by GF-H0.2 with an increment of 26% and GF-H0.1 with 13%. The results also showed that the incorporation of hybrid filler significantly increased the compressive strength by approximately 113% for GF-H0.1, 115% for GF-H0.2, and 95% for GF-H0.3. These increments deduced that the hybrid nanofiller elicited a positive effect and has led to an improvement in the compressive properties of both FRP composites by the synergistic response between the NS and GNP. The mechanical shear force exerted onto the fillers during the fabrication has enhanced the fibre–matrix interphase, which subsequently improves the compressive properties [37].

**Figure 6:**
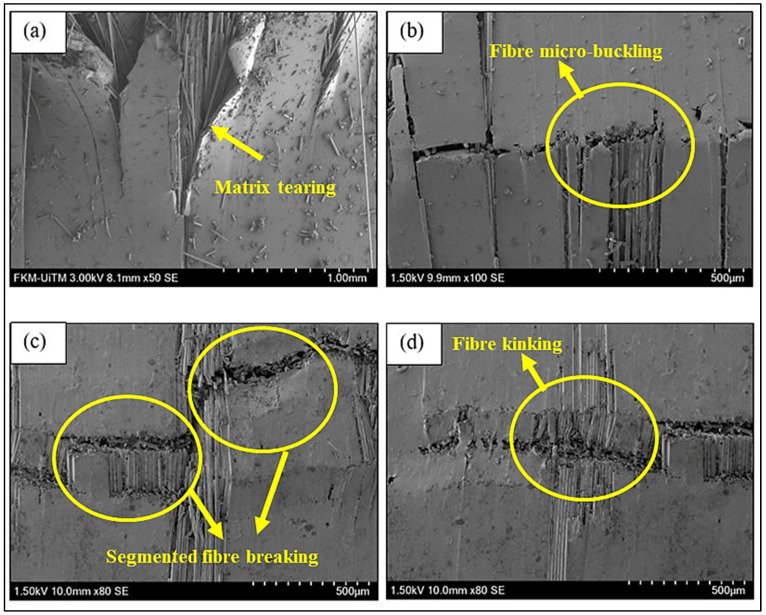
SEM Micrographs of unmodified BFRP composite showing the (**a**) matrix torn and detached from fibre, (**b**) fibre micro buckling, (**c**) matrix cracking and fibre breakage and (**d**) fibre kinking mechanisms.

**Figure 7:**
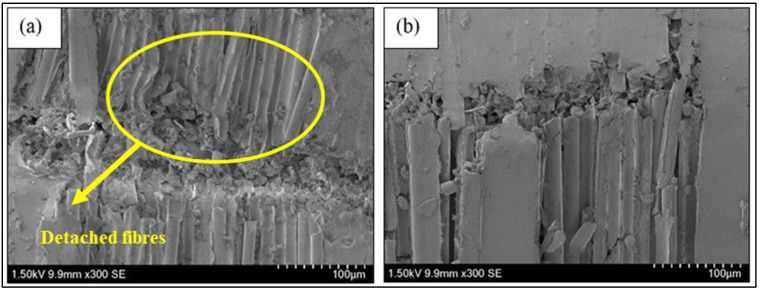
SEM micrographs of fractured (**a**) unmodified BFRP and (**b**) BF-H0.1 composite specimens.

Figure 6 shows the microscopic observations on the post-fracture specimen of BFRP composite observed under SEM. From the observation, it can be seen that the matrix was torn and detached from the fibres in the unmodified BF system as in Figure 6a. The brittle failure of BFRP was observed as the fibre breaks and the matrix cracks along the fractured lines (see Figure 6b). During the test, a cracking sound was observed prior to catastrophic failures due to stored energy released in the specimen. Meanwhile, the failure was also caused by fibres’ instability by segmented fibre breaking as shown in Figure 6c,d, which induced fibre micro buckling and fibre kinking mechanisms [38]. The incorporation of a hybrid nanofiller into the epoxy resin increased the toughness of the epoxy resin under the compressive loading proved by high compressive strength and modulus. The microscopic analysis of the unmodified BF (Figure 7a) shows that the system experienced brittle failure with the presence of crushed fibres and the fibres also detached from the matrix. Meanwhile, BF-H0.1 fractured specimens demonstrated that the specimens experienced brittle failure behaviours with fibre fractures, but remains attached to the matrix (see Figure 7b). This showed that the existence of nanofiller improved the interface bonding between the fibres and matrix as the number of fibres detached from the matrix is low.

#### 3.2.3. Flexural Properties

**Figure 8:**
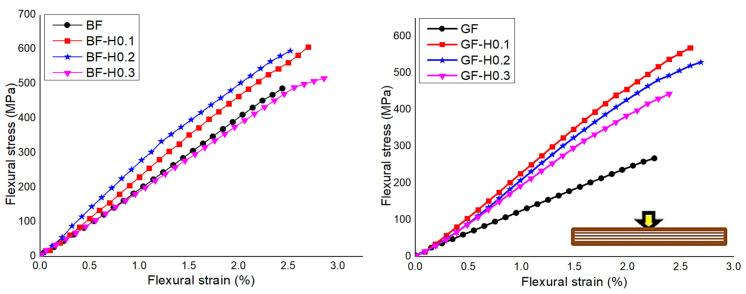
Typical flexural stress-strain response of BFRP and GFRP embedded with hybrid nanofiller.

Figure 8 shows the typical flexural stress-strain curves of FRP composites with a hybrid nanofiller. The effect of NS and GNP combination on the FRP flexural properties are displayed in Table 6. In general, it can be concluded that the incorporation of hybrid nanofiller in both FRP composites resulting in a positive effect on the flexural modulus and strength value. From the results, it can be deduced that the incorporation of 0.1 GNP in the hybrid system of BFRP exhibited the highest flexural strength compared to other systems. The further addition of GNP in the hybrid system has led to a decrease in flexural strength. However, a higher GNP content in the hybrid system resulted in a higher modulus. This is expected as the modulus of GNP is high, and the incorporation of rigid GNP nanofiller together with NS remarkably increase the modulus of the BFRP composites. However, for the GFRP composite, the modulus tends to increase up to GF-H0.2 and started to reduce in GF-H0.3. The flexural strength also tends to increase, but after GF-H0.1, the strength reduced probably due to the existence of distorted GNP, which reduced the reinforcement effect [39]. 

#### 3.2.4. Impact Properties

**Figure 9:**
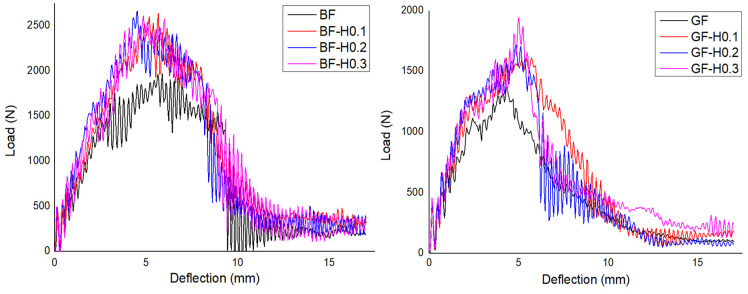
Typical load versus deflection responses of BFRP and GFRP embedded with hybrid nanofiller.

**Figure 10:**
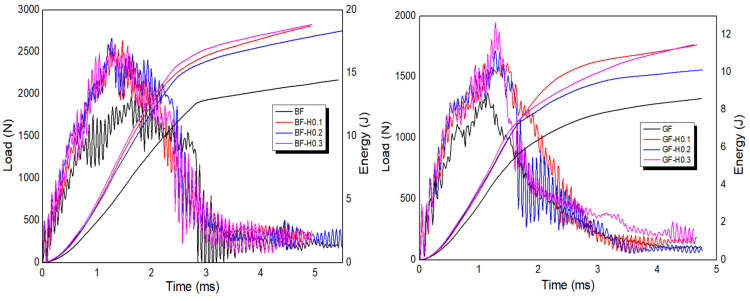
Typical load and energy versus time responses of BFRP and GFRP embedded with hybrid nanofiller.

The effect of filler hybridisation on the FRP impact properties is discussed, as illustrated in Figure 9 and Figure 10. Table 7 shows the results of the impact properties extracted from the graphs. From the results, it was identified that the FRP with hybrid nanofiller has extensively increased their peak load, initiation energy, impact energy and impact strength values. The peak load of BFRP was increased by 32% for BF-H0.1, 4% for BF-H0.2, and 35% for BF-H0.3, respectively, compared to the unmodified BFRP. Meanwhile, GFRP was improved by 16%, 24% and 40% for GF-H0.1, GF-H0.2, and GF-H0.3, respectively, as compared to unmodified GFRP. The initiation energy and impact strength also showed remarkable improvement; even better than with the incorporation of a single nanofiller. This is attributed to the toughening effect of the high modulus and strength nanofiller that firmly hold the fibre resulting in less damage occurring on the specimens. Silica is a ceramic material that is tough and easily dispersed with a proper blending method but is also brittle, while GNP is known for its strength and stiffness but is very challenging to disperse in the epoxy. Hence, the combination of both materials complete the drawback of one another and, in this finding, it has been proved. The incorporation of a single nanofiller already improved the impact properties; however, with hybrid nanofiller, the properties were increased due to the synergistic collaborative effect of GNP and NS [40].

## 4. Conclusions

**Figure 11:**
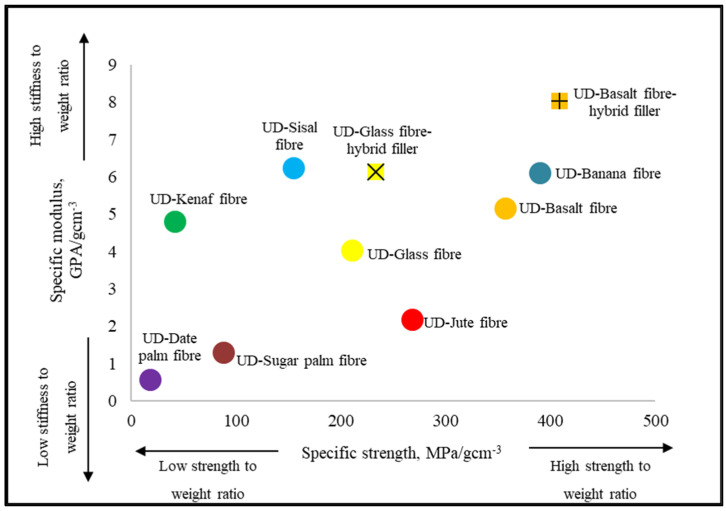
Summary of the specific modulus versus specific strength of different types of natural FRP composites when compared to Glass FRP composites [41,42,43,44,45].

FRP nanocomposites were fabricated using two types of nanofillers; (i) nanosilica (NS) and (ii) graphene nanoplatelets (GNP). The performance of these FRP nanocomposites was evaluated in respect of their mechanical properties. Significant enhancement was observed for the FRP nanocomposites with hybrid nanofiller in tensile, compression, flexural, and impact properties. To summarize, the incorporation of hybrid nanofiller in FRP-H0.2, for both BFRP and GFRP composites showed the optimum performance in most of the tests. This is attributed to less agglomeration and aggregation of the nanofillers that could improve the capabilities and mechanical performance of the FRP system. Hence, this system showed the highest mechanical properties as compared to other systems. The use of basalt fibre as an alternative to glass fibre is found to be effective as the results showed remarkable properties of unidirectional basalt as compared to glass fibre. Figure 11 shows a summary of the specific modulus versus the specific strength of different types of FRP composites obtained from literature and results obtained from this study. When compared to the other natural FRP composites, it is proven that BF is one of the most promising materials as it has high specific stiffness and specific strength. The graph indicated that the existence of hybrid nanofiller in BF and GF significantly improved the specific stiffness of FRP composites, as the density of both materials is low. The specific modulus of BF was 28% higher than GF, while the specific modulus of BF-hybrid was 31% higher than GF-hybrid. Thus, it can be concluded that the incorporation of a hybrid nanofiller improved the mechanical properties of the FRP composites.

## Figures and Tables

**Table 1:** Designation of fabricated specimens.

Label	System	Details
BF	Unmodified basalt fibre composites	Basalt fibre + Unmodified epoxy
BF-NS	BFRP + Nanosilica	Basalt fibre + 25 wt% NS
BF-GNP	BFRP + Graphene nanoplatelet	Basalt fibre + 0.3 wt% GNP
BF-H0.1	BFRP + Hybrid 0.1	Basalt fibre + 0.1 wt% GNP/15 wt%NS
BF-H0.2	BFRP + Hybrid 0.2	Basalt fibre + 0.2 wt% GNP/15 wt%NS
BF-H0.3	BFRP + Hybrid 0.3	Basalt fibre + 0.3 wt% GNP/15 wt%NS
GF	Unmodified glass fibre composites	Glass fibre + Unmodified epoxy
GF-NS	GFRP + Nanosilica	Glass fibre + 25 wt% NS
GF-GNP	GFRP + Graphene nanoplatelet	Glass fibre + 0.3 wt% GNP
GF-H0.1	GFRP + Hybrid 0.1	Glass fibre + 0.1 wt% GNP/15 wt%NS
GF-H0.2	GFRP + Hybrid 0.2	Glass fibre + 0.2 wt% GNP/15 wt%NS
GF-H0.3	GFRP + Hybrid 0.3	Glass fibre + 0.3 wt% GNP/15 wt%NS

**Table 2:** Density of FRP Nanocomposite System Measured by Archimedes Principle.

Nanocomposite Specimen	Density (g/cm^3^)	
	BFRP	GFRP
-Unmodified FRP	1.552 ± 0.043	1.613 ± 0.031
FRP + NS	1.656 ± 0.085	1.697 ± 0.226
FRP + GNP	1.561 ± 0.141	1.625 ± 0.128
FRP + H0.1	1.564 ± 0.157	1.617 ± 0.113
FRP + H0.2	1.564 ± 0.072	1.619 ± 0.265
FRP + H0.3	1.568 ± 0.004	1.620 ± 0.192

**Table 3:** Weight, volume and void volume fraction of fibres and matrices of FRP composite.

	BFRP Composite			GFRP Composite		
**Constituent**	**Epoxy Resin**	**Basalt Fibre**		**Epoxy Resin**	**Glass Fibre**	
**Weight fraction (wt%)**	36.24 ± 4.46	61.15 ± 4.46		32.40 ± 4.34	60.91 ± 4.34	
	**Epoxy Resin**	**Basalt Fibre**	**Void**	**Epoxy Resin**	**Glass Fibre**	**Void**
**Volume fraction (vol%)**	49.78	47.45	2.77	46.25	49.13	4.62

**Table 4:** Tensile properties of FRP embedded with hybrid nanofiller.

System	Tensile Modulus, E_ten_ (GPa)	Ultimate Tensile Strength, σ_ten,u_ (MPa)	Tensile Strain at Break, εt_en,f_ (%)
BF	7.98 ± 1.32	555.96 ± 2.06	4.10 ± 0.15
BF-NS	9.98 ± 2.15	580.94 ± 2.03	3.21 ± 0.23
BF-GNP	10.71 ± 2.43	618.12 ± 1.89	2.90 ±0.11
BF-H0.1	12.45 ± 1.55	643.68 ± 1.21	3.90 ± 0.16
BF-H0.2	13.39 ± 2.32	670.50 ± 2.06	3.00 ± 0.16
BF-H0.3	11.87 ± 2.65	610.34 ± 2.47	3.21 ± 0.22
GF	6.47 ± 1.44	342.56 ± 3.32	3.49 ± 0.12
GF-NS	9.13 ± 2.15	417.12 ± 2.09	3.35 ± 0.22
GF-GNP	8.31 ± 3.55	403.46 ± 2.67	3.11 ± 0.13
GF-H0.1	9.87 ± 2.64	396.68 ± 1.11	2.81 ± 0.13
GF-H0.2	11.72 ± 2.42	384.85 ± 3.14	2.61 ± 0.19
GF-H0.3	8.18 ± 2.55	356.24 ± 3.22	2.67 ± 0.22

**Table 5:** Compressive properties of FRP embedded with hybrid nanofiller.

System	Compressive Modulus, E_comp_ (GPa)	Compressive Strength, σ_comp,u_ (MPa)	Compressive Strain at Break, ε_comp,f_ (%)
BF	5.18 ± 2.06	106.88 ± 1.87	2.30 ± 0.15
BF-NS	6.03 ± 5.43	183.37 ± 2.53	2.10 ± 0.23
BF-GNP	3.90 ± 4.05	148.48 ± 3.03	4.00 ± 0.27
BF-H0.1	8.52 ± 4.11	184.29 ± 2.76	3.02 ± 0.23
BF-H0.2	8.78 ± 4.79	172.13 ± 4.14	2.13 ± 0.22
BF-H0.3	8.15 ± 4.06	178.78 ± 3.05	2.21 ± 0.16
GF	5.04 ± 3.55	77.29 ± 4.32	1.89 ± 0.23
GF-NS	8.04 ± 3.23	137.12 ± 3.39	2.22 ± 0.13
GF-GNP	3.71 ± 5.56	56.50 ± 3.09	4.10 ± 0.12
GF-H0.1	5.72 ± 1.58	165.34 ± 3.24	2.82 ± 0.22
GF-H0.2	6.36 ± 2.08	166.43 ± 3.13	2.73 ± 0.24
GF-H0.3	7.09 ± 2.26	150.56 ± 3.09	2.61 ± 0.15

**Table 6:** Flexural properties of FRP embedded with hybrid nanofiller.

System	Flexural Modulus, E_flex_ (GPa)	Flexural Strength, σ_flex,u_ (MPa)	Flexural Strain at Break, ε_flex,f_ (%)
BF	13.77 ± 3.75	487.08 ± 3.16	2.44 ± 0.25
BF-NS	17.70 ± 3.42	650.08 ± 3.56	2.46 ± 0.13
BF-GNP	15.79 ± 4.65	495.35 ± 3.76	2.22 ± 0.10
BF-H0.1	18.60 ± 4.28	607.54 ± 4.78	2.70 ± 0.32
BF-H0.2	18.74 ± 3.96	595.85 ± 4.09	2.52 ± 0.13
BF-H0.3	18.78 ± 4.07	516.18 ± 3.66	2.86 ± 0.22
GF	8.04 ± 2.21	268.26 ± 4.79	2.25 ± 0.32
GF-NS	12.47 ± 3.11	365.14 ± 2.98	2.06 ± 0.18
GF-GNP	14.02 ± 1.25	446.23 ± 2.76	2.20 ± 0.28
GF-H0.1	14.36 ± 2.64	569.19 ± 3.24	2.59 ± 0.15
GF-H0.2	15.59 ± 3.42	529.78 ± 2.43	2.69 ± 0.19
GF-H0.3	15.16 ± 2.55	443.66 ± 2.72	2.39 ± 0.22

**Table 7:** Impact properties of FRP composites embedded with hybrid nanofiller.

Composite Specimen	BFRP						GFRP					
	BF	BF-NS	BF-GNP	BF-H0.1	BF-H0.2	BF-H0.3	GF	GF-NS	GF-GNP	GF-H0.1	GF-H0.2	GF-H0.3
**Peak load (kN)**	1971.8 ± 0.32	2133.2 ± 0.41	2439.7 ± 0.35	2609.4 ± 0.35	2637.9 ± 0.22	2663.6 ± 0.18	1386.2 ± 0.47	1521.2 ± 0.52	1390.3 ± 0.31	1613.3 ± 0.12	1715.9 ± 0.24	1945.6 ± 0.36
**Deflection at peak load (mm)**	5.878 ± 0.36	5.3364 ± 0.55	4.7077 ± 0.55	5.659 ± 0.64	5.523 ± 0.51	5.844 ± 0.50	6.030 ± 0.32	5.3271 ± 0.12	4.9468 ± 0.38	5.712 ± 0.22	4.852 ± 0.16	5.007 ± 0.17
**Initiation energy, E_m_ (J)**	5.680 ± 3.32	9.3314 ± 3.34	8.5862 ± 3.65	6.465 ± 4.32	7.231 ± 4.30	8.023 ± 4.01	5.145 ± 4.32	5.3632 ± 6.15	3.8407 ± 2.76	5.261 ± 5.23	5.765 ± 5.12	5.832 ± 4.98
**Propagation energy, E_p_ (J)**	7.183	8.3929	13.3712	12.560	12.627	12.967	3.363	7.9640	5.6989	9.360	10.284	9.530
**Impact energy (J)**	42.428 ± 0.12	47.4452 ± 0.19	46.3972 ± 0.42	47.344 ± 0.11	49.549 ± 0.20	52.100 ± 0.19	35.835 ± 0.06	43.3272 ± 0.52	36.2178 ± 0.32	39.887 ± 0.24	42.179 ± 0.34	43.239 ± 0.34
**Ductility index, DI**	1.26	0.90	1.56	1.94	1.75	1.62	0.65	1.49	1.48	1.82	1.78	1.63
**Impact strength (kJ/m^2^)**	35.726	41.747	42.704	62.468	62.801	64.492	16.726	39.610	19.102	46.556	51.148	47.401
